# MicroRNA-Directed Biomarkers and Breast Cancer Therapeutics—Potential to Advance Personalised Approaches in Clinical Trials

**DOI:** 10.3390/ijms27093996

**Published:** 2026-04-29

**Authors:** Luis Bouz Mkabaah, Eoin P. Kerin, Matthew G. Davey, Eleftheria Filandrianou, Vinitha Richard, Michael J. Kerin

**Affiliations:** 1 Department of Surgery, The Lambe Institute for Translational Research, University of Galway, H91 YR71 Galway, Ireland; l.bouzmkabaah1@universityofgalway.ie (L.B.M.);; 2 Royal College of Surgeons in Ireland, 123 St. Stephens Green, Dublin 2, D02 YN77 Dublin, Ireland

**Keywords:** breast cancer, microRNAs, personalised care, molecular oncology, precision oncology

## Abstract

The advent of breast cancer molecular subtyping has transformed management, enabling treatment personalisation and de-escalation beyond traditional stage-based approaches. Established biomarkers, such as Ki-67 in luminal disease, HER2 amplification, and PD-L1 expression in triple-negative breast cancer, underpin seminal clinical trials yet remain imperfect predictors of response and long-term outcome. MicroRNAs have emerged as promising next-generation biomarkers and therapeutic tools. As master regulators of gene expression, both tumour-derived and circulating microRNAs can refine diagnosis and molecular subclassification, inform prognosis and therapeutic selection, act as treatment sensitisers, and potentially serve as direct therapeutic targets. Well-characterised miRNAs such as miR-221 have been implicated in endocrine resistance, while recent liquid-biopsy approaches have enabled the identification of circulating miR-145 and exosomal miR-155 as predictors of pathological complete response in HER2-positive disease. Their detectability in tissue, blood and other biofluids offers a minimally invasive means to dynamically monitor cancer behaviour and response, supporting more precise therapeutic decision-making. This review synthesises the current evidence for miRNA-based biomarkers across oestrogen-receptor positive, HER2-positive and triple-negative breast cancer and outlines their potential integration into biomarker-driven clinical trial designs and personalised treatment strategies.

## 1. Introduction

Breast cancer is the most frequently diagnosed cancer among women and ranks as the second leading cause of cancer-related deaths [1]. While surgery remains the cornerstone of breast cancer management, contemporary treatment strategies encompass systemic chemotherapy, radiotherapy, endocrine therapy, targeted therapy and related modalities. More recently, improved understanding of the molecular pathology of breast cancer has enabled increasingly personalised therapeutic approaches, enhancing treatment efficacy while minimising toxicity [2]. Although these novel therapeutic strategies have resulted in notable advancements in patient survival and quality of life, accurately predicting how each patient will respond and stratifying individual risk of recurrence and long-term prognosis remain a challenge.

Clinical trials have been pivotal in shaping the contemporary breast cancer treatment paradigm: trials such as TAILORx, RxPONDER and MINDACT have demonstrated how genomic expression assays may have clinical utility in guiding therapeutic decision-making, leading to an overall reduction in chemotherapy prescription, without compromising oncological and survival outcomes [3,4,5]. MicroRNAs (miRNAs or miRs) may have future applicability in these roles.

**Figure 1:**
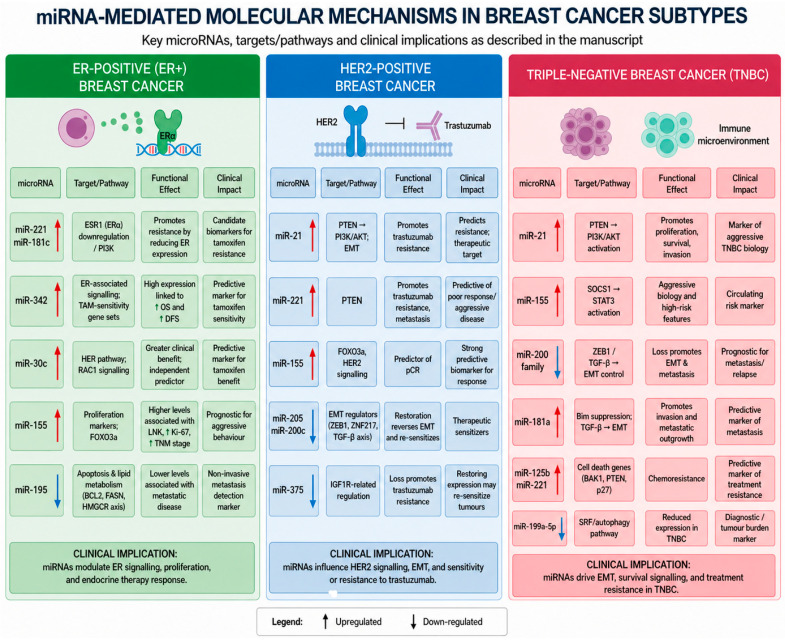
Schematic overview of selected microRNAs and their associated targets, pathways, and clinical implications across breast cancer subtypes.

First described by Lee et al. in 1993 through studies on the nematode *Caenorhabditis elegans* [6], miRNAs are small non-coding RNAs of approximately 19 to 25 nucleotides in length that regulate gene expression and have shown considerable promise as both diagnostic and prognostic biomarkers in breast cancer [7]. Despite promising results from lab-based and exploratory retrospective analyses, the use of miRNA profiling in clinical practice remains a work in progress. miRNAs are detectable across a wide range of biological sources, including tumour tissue, whole blood, serum, plasma, and other body fluids, with distribution varying substantially between these compartments [8]. There is increasing interest in their ability to improve prediction of treatment response, refine risk assessment, and guide treatment de-escalation strategies. The functional utilisation and integration of miRNAs into clinical practice are underpinned by their role as master regulators of gene expression, thus enabling wide-ranging biological influence and potentially multifunctional clinical applicability [7]. A schematic overview of key microRNA-mediated pathways across breast cancer subtypes is provided in Figure 1, while a more comprehensive summary of reported microRNAs and their clinical associations is presented in Table 1, Table 2 and Table 3.

## 2. Endocrine Receptor-Positive Disease

### 2.1. Clinical Characteristics and Implications

Approximately 70% of breast cancers are classified as endocrine responsive, based on the expression of oestrogen receptor (ER), progesterone receptor (PR), or both [9]. Major breakthroughs in the understanding of ER-positive (ER+) breast cancer have revolutionised the treatment of the disease. While surgery followed by endocrine therapy remains the foundation of ER+ breast cancer treatment, advancements in targeting non-hormonal pathways, such as cyclin-dependent kinase 4/6 (CDK4/6), mammalian target of rapamycin (mTOR), protein kinase B (Akt), and phosphatidylinositol 3-kinase (PI3K), have led to the development of novel therapies that have significantly improved patient outcomes [10]. Despite these advancements, resistance to endocrine treatment remains a challenge in the management of advanced ER+ breast cancer [9]. miRNAs, which play critical roles in post-transcriptional gene regulation, have been implicated in modulating the signalling pathways associated with endocrine receptors. Their theoretical potential as biomarkers for diagnosis, prognosis, and treatment response, as well as their role in resistance to endocrine therapy, supports their investigation as candidate tools to refine therapeutic stratification and improve patient outcomes [11].

### 2.2. ER+ Disease Fundamentals: Ki-67

It is well established that ER+ breast cancer is associated with a favourable prognosis when compared to HER2-positive (HER2+) breast cancer and triple-negative breast cancer (TNBC) [12]. This can be predicted using antigen Ki-67, a protein encoded by the MKI67 gene that acts as a marker of cellular proliferation [13]. Ki-67 has been used as a tool to predict long-term outcomes in breast cancer patients for decades [14], with its use in luminal A/B molecular subtype stratification and its implementation in the Preoperative Endocrine Prognostic Index (PEPI) score, underscoring its prognostic utility. As such, Ki-67 has been incorporated into numerous clinical trials, ratifying its role in the current breast cancer paradigm [15,16,17]. Despite its utility, Ki-67 is limited by inconsistencies in assessment methodology and poor interobserver reproducibility, meaning standardisable biomarkers may potentially offer a complementary or superior means of risk stratification in this setting [18].

Several studies have examined the relationship between Ki-67 expression and miRNA dysregulation in an effort to capture their diagnostic, therapeutic, and prognostic potential, albeit not stratified by molecular subtype [19,20]. For example, increased stromal miR-20a-5p expression correlates with higher Ki-67 levels and more aggressive clinicopathological features [21]. Furthermore, overexpression of tissue-based miR-21 and miR-10b, and both plasma and tissue miR-155 levels, has been associated with a higher Ki-67 index [21,22,23,24,25]. These findings suggest that miRNAs capture proliferative biology reflected by Ki-67 but with the added advantages of greater analytical standardisation and minimal invasiveness, with potential integration into adaptive clinical trial designs. Collectively, this underscores the promise of miRNA-driven biomarkers in refining endocrine response prediction, improving risk stratification, and informing personalised treatment strategies for ER+ disease beyond the limitations of Ki-67 alone.

### 2.3. Current Value of miRNAs in Endocrine-Responsive Disease

Several miRNAs have shown strong promise as predictive and prognostic biomarkers, with many miRNA signatures linked to endocrine therapy resistance. Most available data derive from preclinical models and retrospective cohort analyses, with relatively limited prospective validation to date. A study by Healy et al. identified two tissue-based miRNAs, miR-221 and miR-181c, which were upregulated in tamoxifen resistance, directly contributing to loss of ER activity and worse response to endocrine therapy through ERα downregulation. MiR-181c was demonstrated to regulate the PI3K pathway along with ER signalling, where its overexpression was associated with resistance to tamoxifen and worse survival [26]. Other miRNAs have been linked to ERα downregulation, including miR-342–3p, miR-873 and Let-7b/Let-7i [27]. Furthermore, miR-375 has been shown to reverse both tamoxifen resistance and its accompanying epithelial-to-mesenchymal transition (EMT) [28].

miRNAs have been identified as markers of sensitivity to endocrine therapy, further enhancing their prognostic value. Cittelly et al. demonstrated miR-342–3p to be downregulated in tamoxifen-resistant tumour cell lines. They also found higher expression levels to correlate with longer disease-free survival (DFS) in tamoxifen-treated ER+ patients [29]. Through multivariate analysis, Rodriguez et al. identified tissue-based miR-30c to be an independent predictor for clinical benefit in tamoxifen therapy by modulating RAC1 signalling pathways [30]. Upregulation of miR-155 has been linked to metastasis in primary breast cancer, particularly in patients with ER+ breast cancer, through promoting invasion, migration and EMT [31]. The diagnostic role of miR-195 in breast cancer was elucidated by Heneghan et al. in 2010 [32]. Further work in the field of ER+ breast cancer identified miR-195 as a tumour-suppressive miRNA, with downregulation of tumour and circulating miR-195 associated with higher proliferation, adverse outcomes and metastatic disease [33,34]. The majority of studies investigating the role of miRNAs in ER+ breast cancer to date encompass predominantly preclinical and retrospective evidence, highlighting a significant gap and opportunity for translational research to validate and implement miRNA-based biomarkers in prospective clinical settings.

### 2.4. Clinical Trials

In a retrospective analysis of large-scale trial-associated cohorts, increased miR-342 expression was significantly associated with better overall survival (OS) and DFS across The Cancer Genome Atlas (TCGA) and multiple Gene Expression Omnibus (GEO) datasets, particularly among ER+ breast cancer patients undergoing tamoxifen therapy [35]. For example, absence of miR-451 in tumour tissue or circulating samples could help identify patients who, despite appearing low-risk at the genomic level, may still have a functionally aggressive disease and could benefit from chemotherapy or additional treatments like CDK4/6 inhibitors [36]. Conversely, patients with favourable miRNA profiles might qualify for shorter durations of endocrine therapy or omission of axillary surgery, as explored in trials like SOUND and INSEMA [37,38]. To date, these hypotheses remain largely exploratory and are not yet incorporated into randomised trial stratification.

Regarding prospective evidence, tumour-based miR-100 was evaluated in a study of 90 breast cancer patients undergoing endocrine therapy, where higher baseline miR-100 was independently associated with a greater likelihood of endocrine response and subsequently formed the basis of an miR-100-target gene signature that outperformed Ki-67 alone in predicting the luminal A phenotype [39]. This provides early translational evidence that miRNAs have the potential to serve as practical clinical prognostic biomarkers that may perform comparably to or outperform traditional pathological measures. Other newer observational cohort studies, such as the BREMIR study, aim to validate tissue and plasma miRNA panels prospectively. In its discovery cohort, eight miRNAs were linked to metastatic progression, with miR-3916 and miR-3613–5p emerging as the strongest discriminators between metastatic and non-metastatic tumours. In the extended validation cohort (*n* = 223), these two miRNAs retained independent prognostic value and, when incorporated into a multivariable model alongside stage, grade, and Ki-67, significantly improved metastatic-risk prediction (AUC 0.85 vs. 0.76 for clinicopathological variables alone). However, while miRNAs have been evaluated within large prospective clinical trials in an exploratory and translational capacity, they have not yet been incorporated as primary endpoints nor implemented as mandatory stratification factors to guide treatment assignment in standard clinical practice [40]. This represents a key translational gap between biomarker discovery and clinical implementation. Future work in this field will require biomarker-driven clinical trials that assess whether miRNA signatures can meaningfully refine therapeutic decision-making in ER+ disease. miRNAs linked with ER+ disease and their expression, targets and clinical implication are available in Table 1.

**Table 1:** miRNAs linked with ER+ breast cancer.

miRNA	Tissue	Expression Change	Target/Pathway	Effect/Functional Note	Clinical Implication	Author (Year)
miR-10b	Tumour	↑ with stage; ↑ in LN+ and high Ki67	Migration/invasion pathways	Higher levels linked to worse stage, LN+, larger tumours	Prognostic for aggressive biology	[23]
miR-20a-5p	Tumour	↑ in stroma/cytoplasm	miR-17–92 → proliferation/migration	Higher levels linked to ↑ Ki67 and ↑ relapse; promotes migration/invasion	Prognostic for aggressive biology	[21]
miR-21	Tumour	↑ vs. adjacent tissue	PTEN → PI3K/AKT	Higher levels linked to ↑ TNM stage, lymph node positivity, ↑ Ki67; no link to ER status	Prognostic for aggressive features	[22]
miR-30c	Tumour	↑ in tumours from patients benefiting from tamoxifen	HER pathway; RAC1 cell-motility signalling	Higher levels linked to ↑ PFS and greater clinical benefit; independent predictor of TAM response	Predictive marker for tamoxifen benefit	[30]
miR-100	Tumour	↑ in endocrine-responsive tumours	PLK1, FOXA1, mTOR, IGF1R (all ↓ when miR-100 ↑)	Higher levels linked to ↓ baseline and post-treatment Ki67; ↑ chance of complete cell-cycle arrest; defines less aggressive, endocrine-sensitive biology	Predictive of aromatase inhibitor response; prognostic in luminal A	[39]
miR-155	Tumour/plasma	↑ in BC patients vs controls	OncomiR; proliferation markers (Ki67/p53 associations), FOXO3a; proliferation/anti-apoptosis	Higher levels associated with LN+, ↑ Ki67, ↑ TNM stage; higher in ER+ and PR+ tumours	Prognostic for aggressive behaviour	[24,25]
miR-155	Tumour	↑	ZNF652, SOCS1 → pro-invasion/EMT	Promotes invasion and metastasis in ER+ disease	Pro-metastatic; no predictive role	[31]
miR-181-c, miR-221	Tumour	↑ in tamoxifen-resistant cells	ESR1 (ERα) downregulation/suppression, PI3K	Promotes resistance by reducing ER expression	Candidate biomarkers for tamoxifen resistance	[26]
miR-195	Plasma/whole blood	↓ in metastatic vs local luminal A	Apoptosis and lipid metabolism (BCL2, FASN/HMGCR axis)	Lower levels associated with metastatic disease; acts as tumour suppressor	Non-invasive metastasis detection marker	[34]
miR-342	Tumour	↑ miR-342 = favourable biology	ER-associated signalling; TAM-sensitivity gene sets	High expression linked to ↑ OS and ↑ DFS; enriched TAM-sensitivity genes; low expression enriched TAM-resistance genes	Strong prognostic and predictive marker for TAM sensitivity in ER+ disease	[35]
miR-342–3p	Tumour	↓ in tamoxifen-resistant cells and ↓ in TamR patient tumours	EVL/ER-associated regulation; apoptosis and cell-cycle genes	Loss promotes TAM resistance; restoring miR-342 re-sensitises cells and ↑ apoptosis	Predictive marker for TAM response; candidate re-sensitisation target	[29]
miR-342–3p, miR-873, let-7b/let-7i	Tumour	All ↓ in tamoxifen-resistant cells	ERα expression/ERα36 variant regulation	Loss of these miRNAs reduces ERα signalling and promotes tamoxifen resistance; restoring them re-sensitises cells to TAM	Potential predictive markers of endocrine resistance; candidates for therapy re-sensitisation	[27]
miR-375	Tumour	↓ strongly downregulated in TamR cells	MTDH (metadherin) → EMT/invasion	Loss of miR-375 drives TAM resistance and EMT-like phenotype; re-expression restores TAM sensitivity and reduces invasion	Biomarker of TAM resistance; therapeutic re-sensitisation target	[28]
miR-3916	Tumour/plasma	↑ in metastatic vs non-metastatic breast tumours	—	Strongly discriminates metastatic from non-metastatic disease; remained significant in multivariable analysis	Independent prognostic marker for metastatic progression; improves risk prediction when added to clinicopathological variables	[40]
miR-3613–5p	Tumour/plasma	↑ in metastatic vs non-metastatic breast tumours	—	Distinguishes metastatic from non-metastatic cases; remains independently associated with relapse in expanded cohort	Prognostic biomarker of metastatic risk; part of high-performing model	[40]
miR-451	Tumour/plasma	Absent/↓ in aggressive disease	MDR1/stress-response pathways	Loss of miR-451 associated with more invasive biology; absence may signal functionally high-risk disease even when genomic markers look low risk	Low/absent miR-451 may help identify patients who could benefit from chemotherapy or CDK4/6 inhibitors despite low-risk genomic profiles	[36]

↑; upregulated, ↓; downregulated.

## 3. HER2-Positive Disease

### 3.1. Clinical Characteristics and Implications

Human epidermal growth factor receptor 2-positive (HER2+) breast cancer represents approximately 20–25% of all diagnosed breast cancer cases [41]. Overexpression of the HER2 gene in breast cancer is associated with aggressive tumours and poorer clinical outcomes [42]. Building on their discovery of the HER2 gene, Slamon et al. played a pivotal role in transforming the treatment landscape of HER2+ breast cancer through the development and introduction of anti-HER2 targeted therapies, such as trastuzumab, a breakthrough that remains a benchmark drug in contemporary targeted therapeutics [43]. Emerging preclinical and translational evidence indicates that dysregulated miRNAs contribute to HER2 tumour biology and may influence prognosis and response to HER2-targeted therapy.

### 3.2. HER2 and Pathological Complete Response (pCR)

Pathological complete response (pCR) is defined as the absence of invasive or in situ residual disease [44]. Specific to HER2+ breast cancer, there is substantial clinical evidence supporting pCR as a surrogate marker for improved survival outcomes [45,46]. In accordance with the most recent American Society of Clinical Oncology/College of American Pathologists (ASCO/CAP) guidelines (2021), all patients diagnosed with HER2+ breast cancer should be considered for neoadjuvant chemotherapy (NAC) to maximise pCR rates (with exceptions limited to those with cT1a/b/N0 disease) [47]. A study conducted by Gianni et al. examined the relationship between intrinsic breast cancer subtypes and pathological complete response (pCR) in patients with HER2+ disease receiving neoadjuvant therapy (NAT), finding that the HER2+ subtype demonstrated higher pCR rates compared to other subtypes, irrespective of hormone receptor status [48]. Several miRNAs, including circulating miR-145, plasma miR-140–5p, and exosomal miR-155 and miR-301, have been shown in major neoadjuvant trials (NeoALTTO, ICORG 10/11, GeparQuinto, GeparSixto) to predict pCR in HER2+ breast cancer [49,50,51,52].

### 3.3. Trastuzumab

Trastuzumab, or Herceptin^®^, is a monoclonal antibody (mAb) that targets domain IV on the extracellular portion of HER2, suppressing its signalling and effect on protein transcription [53]. The NOAH trial, a prospective phase III trial comparing NAC plus Trastuzumab to NAC alone, demonstrated significantly improved rates of pCR and event-free survival (3-year event-free survival, 71% [95% CI 61–78; *n* = 36 events] with trastuzumab vs 56% [46–65; *n* = 51 events] without; hazard ratio 0.59 [95% CI 0.38–0.90]; *p* = 0.013) [48]. Multiple other randomised controlled trials (RCTs) that investigated the effects of combining Trastuzumab with chemotherapeutic agents demonstrated significantly improved survival outcomes [54,55,56]. Further molecular understanding of HER2+ breast cancer has allowed for the introduction of dual anti-HER2 therapy in combination with chemotherapy in the neoadjuvant setting, allowing for even higher pCR rates [57,58]. Further combinations, such as trastuzumab emtansine (T-DM1), an antibody–drug conjugate of trastuzumab and the cytotoxic agent emtansine (DM1), demonstrated lower recurrence rates in patients with residual HER2+ disease following neoadjuvant treatment [59]. Sequential findings of the EMILIA and KATHERINE trials also supported the benefit of T-DM1 [60,61]. miRNAs such as miR-21, miR-221, miR-1246 and miR-155 have been shown to promote trastuzumab resistance, whereas miR-205, miR-200c and miR-375 restore sensitivity, supporting their role as biomarkers of trastuzumab response [49,62,63,64,65].

### 3.4. Current Value of miRNAs in HER2+ Disease

There is growing evidence that specific miRNAs play a key role in regulating the HER2 signalling cascade, affecting how tumours respond to trastuzumab, and impacting critical downstream processes such as EMT, immune system evasion, and cell survival pathways [66]. Several miRNAs have been extensively studied in HER2+ breast cancer and have been linked to treatment resistance, sensitivity modulation and prognosis via preclinical and retrospective analyses, with limited prospective clinical validation to date [67]. MiR-21 has been identified as a central mediator for trastuzumab resistance. Gong et al. suggested that downregulation of miR-21 was linked to trastuzumab resistance primarily through downregulating the tumour suppressor PTEN, leading to persistent PI3K/AKT signalling and therapeutic failure [68]. Furthermore, miR-21 has been shown to sustain EMT and shape the tumour immune microenvironment in the setting of HER2-positive breast cancer [66]. Inhibiting miR-21 using antisense oligonucleotides can restore trastuzumab sensitivity both in vitro and in vivo, highlighting its promise as a biomarker and potential therapeutic target [69]. Other oncogenic miRNAs have been implicated in resistance. The work of Ye et al. linked miR-221 with aggressive HER2 disease features and demonstrated that its overexpression is related to trastuzumab resistance and metastatic traits through direct targeting of PTEN [63]. Additionally, upregulation of miR-1246 and miR-155 has been linked to trastuzumab-resistant HER2+ breast cancer [64]. Notably, miR-375 has been found to be frequently epigenetically silenced in trastuzumab-resistant tumours due to IGF1R-driven chromatin remodelling, leading to uncontrolled HER2 signalling and therapeutic escape [70]. Restoration of miR-205 and miR-200c expression has been shown to reverse resistance by inhibiting epithelial-to-mesenchymal transition (EMT), reducing invasive capacity and re-sensitising HER2+ cells to trastuzumab [65,71]. Other studies aimed to assess the role of miRNAs in predicting response to NAT to achieve pCR in HER2+ breast cancer patients. A systematic review concluded that upregulation of 41 miRNAs and downregulation of 29 miRNAs predicted differential response to NAT. Among these, miR-221 was reported to be upregulated in trastuzumab-resistant HER2-positive disease and is associated with poorer pCR rates. Conversely, miR-222–3p is typically downregulated in patients who achieve pCR, suggesting a role as a favourable predictive marker. miR-205, assessed in tumour tissue, is upregulated in trastuzumab responders and may enrich for patients most likely to benefit from HER2-targeted therapy. Finally, miR-199a shows response-associated dysregulation across cohorts and may contribute to multi-miRNA signatures predictive of therapeutic sensitivity [72]. miRNA signatures hold potential clinical value in helping to predict patient outcomes, inform treatment response, and enhance existing therapeutic approaches.

### 3.5. miRNA Clinical Trials

Many translational studies conducted alongside key HER2-targeted neoadjuvant trials have explored the role of miRNAs as early indicators of how well patients respond to treatment, especially in achieving pCR. In the NeoALTTO trial, Di Cosimo et al. reported that patients who achieved a pCR after two weeks of trastuzumab treatment showed elevated levels of plasma miR-145. Additionally, lower miR-145 expression levels were observed in non-responders compared to responders following neoadjuvant chemotherapy (*p* = 0.033) [52]. Findings regarding the role of miR-145 were replicated in the multicentre ICORG 10/11 trial [51]. The NeoALTTO trial further identified an early circulating miRNA signature measured at two weeks, including miR-140-5p, that predicted pCR to trastuzumab monotherapy, with an area under the curve (AUC) of 0.81, independent of hormone receptor status. A later tissue-based analysis identified baseline miRNAs such as miR-153-3p and miR-219a-5p, which correlated with both pCR and event-free survival [52]. The GeparQuinto trial by Muller et al. found that elevated baseline levels of miR-21 were able to distinguish responders from non-responders in HER2+ breast cancer [49]. In the GeparSixto trial of triple-negative and HER2+ breast cancer exploring the impact of adding carboplatin-based chemotherapy, exosomal miRNA profiling in 211 HER2+ patients identified a panel of deregulated miRNAs. Exosomal miR-155 and miR-301 emerged as independent predictors of pCR in multivariate analysis [50]. Despite these promising prospective findings, miRNAs are not yet used to guide trial stratification or treatment decision-making in HER2+ disease, highlighting an important translational gap. miRNAs linked with HER2+ disease are available in Table 2.

**Table 2:** miRNAs linked with HER2+ breast cancer.

miRNA	Tissue	Expression Change	Target/Pathway	Effect/Functional Note	Clinical Implication	Author (Year)
miR-21	Tumour	↑ in resistance	PTEN → PI3K/AKT; EMT; immune TME	Promotes trastuzumab resistance, EMT, immune changes	Predicts resistance; therapeutic target (anti-miR-21)	[68]
miR-21	Serum	↑ in patients vs. healthy controls; further ↑ after neoadjuvant therapy	PTEN axis; apoptosis-related signalling	Levels rise with chemotherapy; no association with pCR; higher baseline and post-therapy levels predict	Prognostic marker (poor OS)	[49]
miR-140a-5p	Plasma	↑ in early trastuzumab responders	Putative tumour-suppressive pathways; predicted regulation of apoptosis, cell-cycle, Wnt pathway	Higher levels associated with greater chance of pCR and significantly better EFS	Predictive biomarker for trastuzumab; potential prognostic value; supports treatment de-escalation decisions	[52]
miR-145	Plasma	↑ in early trastuzumab responders	—(response-associated signature)	Higher plasma levels at 2 weeks associated with pCR	Early circulating predictor of pCR to trastuzumab	[51,52]
miR-148a-3p	Plasma	↑ in trastuzumab responders	Not assessed in study; literature: regulates angiogenesis (NRP1)	Early increase (2 weeks into therapy) associated with pCR	Part of multi-miRNA pCR predictor for trastuzumab	[52]
miR-155	Exosomal plasma	↑ vs. healthy controls; ↓ after neoadjuvant therapy	FOXO3a, HER2 signalling, immune/inflammatory pathways	Most significant predictor of pCR	Strong predictive biomarker for pCR to neoadjuvant therapy (including carboplatin responses); dynamic marker of treatment effect	[50]
miR-155	Plasma	↑ in resistance	Immune/JAK-STAT; EMT	Associated with resistance and metastasis; dynamic changes post-therapy	Circulating biomarker for response; prognostic	[64]
miR-199a	Plasma	Aberrant expression associated with pCR	Tumour-suppressive (mTOR, MET)	Associated with pCR in GeparSixto HER2 cohort; inconsistent direction but strong signal	Potential component of HER2+ predictive panel	[50]
miR-205/miR-200c	Tumour	↓ in resistant tumours	EMT regulators (ZEB1, ZNF217, TGF-β axis)	Restoration reverses EMT and re-sensitises to trastuzumab	Therapeutic sensitisers; prognostic for metastasis risk	[65]
miR-210	Serum	↑ vs. healthy controls; ↑ further after chemotherapy	Hypoxia/HIF-1α pathway	Elevated pre- and post-therapy; no association with pCR or survival	No predictive or prognostic utility	[49]
miR-221	Tumour	↑ in resistance	PTEN	Promotes trastuzumab resistance, metastasis	Predictive of poor response/aggressive disease	[63]
miR-301	Exosomal plasma	↑ vs. healthy controls; stable pre/post therapy	PTEN/Akt, NF-κB, ER pathway modulation	Strong independent predictor of pCR	Predictive exosomal biomarker for response to neoadjuvant therapy (especially carboplatin-based) in HER2+ BC	[50]
miR-373	Serum	↑ vs. healthy controls; ↑ further after chemotherapy	Metastasis/invasion-related pathways	Higher levels associated with advanced clinical tumour stage; no association with pCR or survival	Marker of advanced disease	[49]
miR-374a-5p	Plasma	↑ in trastuzumab responders	Not assessed; literature: oncomiR promoting proliferation via ARRB1/AKT	Rise at week 2 linked to pCR to trastuzumab	Early dynamic predictor of HER2-targeted therapy response	[52]
miR-375	Tumour	↓/epigenetically silenced in resistant tumours	IGF1R-related epigenetic regulation	Loss promotes trastuzumab resistance	Restoring expression may re-sensitise tumours	[63]
miR-1246	Plasma	↑ in resistance	Oncogenic signalling (circulating)	Associated with trastuzumab resistance; circulating biomarker	Candidate circulating predictor of poor response	[64]

↑; upregulated, ↓; downregulated.

## 4. Triple-Negative Disease

### 4.1. Clinical Characteristics and Challenges

Triple-negative breast cancer (TNBC) is characterised by the absence of ER, PR and HER2 receptor activity [73]. While accounting for only 10–15% of all breast cancer diagnoses, TNBC contributes to approximately 40% of breast cancer-related mortality [74]. Clinically, TNBC has a propensity for high grade and proliferation capacity, with increased likelihood of early visceral metastasis and presentation at an advanced or inoperable disease stage [75]. The TNBC disease spectrum is characterised by marked heterogeneity in disease behaviour and response. Lehmann et al.’s seminal transcriptomic analysis identified six TNBC intrinsic molecular subtypes, later refined by Burstein et al. into four: androgen receptor positive (LAR), mesenchymal (MES), basal-like immune activated (BLIA), and basal-like immune suppressed (BLIS) [76,77]. Approximately 75% of TNBCs are basal-like (BLIA/BLIS), such that the terms ‘basal-like’ and ‘TNBC’ are often used synonymously in the literature [78], herein creating further diagnostic and therapeutic uncertainty [77].

Relative to other molecular subtypes, there is a paucity of targeted therapies in TNBC, making surgical resection combined with systemic chemotherapy the main therapeutic option. However, response durability is often limited, and long-term outcomes remain inferior compared to other subtypes [79]. Systemic toxicity and a developing chemoresistance profile compound such therapeutic challenges [80]. Despite these challenges, altered biology and unpredictable therapeutic response patterns, TNBC is typically managed as a single entity in clinical practice, underscoring the need for robust and reproducible biomarkers to guide therapy through the ascertainment of therapeutically distinct intrinsic molecular subtypes [81].

### 4.2. Principles—Treatment Response, pCR, and Prognostic Implications

With the exception of those with early-stage node-negative disease, NAC is considered the first-line treatment modality in TNBC consensus guidelines [82], making measures of response to preoperative therapies crucial for patient prognostication. As previously defined, pCR is widely accepted as the gold-standard surrogate marker for long-term outcome following NAT in aggressive breast cancer subtypes. TNBC exhibits high pCR rates that are strongly predictive of improved DFS and OS [44]. Accordingly, pCR has been adopted as a primary endpoint in several landmark TNBC trials. GeparSixto [50], CALGB 40603 (Alliance) [83], BrighTNess [84], KEYNOTE-522 [85], and IMpassion031 [86] demonstrated that incorporating platinum agents, PARP inhibitors, or immune checkpoint blockade into NAT regimens can significantly increase pCR rates. While pCR is accepted as an excellent surrogate marker for long-term outcomes in TNBC, the I-SPY 1 TRIAL [87] of Esserman et al. surmised that the extent of the benefit that pCR confers varies significantly based on molecular subtype. Subsequent analyses by Prat et al. suggested pCR predicts long-term outcomes primarily in basal-like TNBC alone but inconsistently in other subtypes [88], highlighting ongoing prognostic and therapeutic uncertainty and the necessity for more refined molecular biomarkers of response and further subtype refinement.

### 4.3. Current Value of miRNAs in TNBC

miRNAs have emerged as potential indicators to refine subclassification, improve diagnostic precision, inform treatment strategies, and act as therapeutic sensitisers or targets in TNBC [89]. Preclinical evidence indicates that miRNAs contribute to TNBC pathogenesis through modulation of key molecular signalling pathways. For instance, miR-21 (elevated in both blood and tissue of patients with breast cancer) is associated with reduced PTEN expression, promoting increased cell turnover and invasiveness [90,91]. Tissue miR-155 upregulates the JAK/STAT pathway and is linked to BRCA1 dysfunction, conferring a more aggressive phenotype [92,93]. Furthermore, elevated serum miR-155 has been proposed as a potential non-invasive biomarker for BRCA1 mutation-associated TNBC [94]. miR-498 expression is decreased in BRCA1-associated TNBC cells [95], while a broad array of miRs have been implicated in the p53 signalling pathway [96].

Beyond tumourigenesis, miRNAs also correlate with disease stage and progression. miR-126–5p and miR-135b-5p tissue expression correlates linearly with tumour size [97], while TGF-β-induced upregulation of miR-181a has been shown to promote invasion and metastatic outgrowth in TNBC through EMT [98]. Similarly, members of the miR-200 family (miR-200b, miR-200c, miR-205) are involved in EMT, with reduced tissue expression predicting disease spread through lymph node metastasis, late relapse, and poorer DFS [99,100]. Pertaining to liquid biopsy specifically in the translational setting, increased miR-200c levels predicted relapse regardless of breast cancer subtype along with miR-21 in a study by Papadaki et al. [101], while dynamic fluctuations in serum miR-200 levels during disease progression and post-treatment further suggest a role as circulating biomarkers of treatment response across breast cancer [102]. Further evidence from Shin et al. proposes miR-21, miR-16, and, particularly, miR-199a-5p as TNBC-specific circulating biomarkers, with all three markedly suppressed in plasma of TNBC patients and restored post-tumour removal, highlighting their potential utility for minimally invasive diagnosis and tumour-burden assessment [103].

A 2022 systematic review by Santana et al. summarised evidence as to the prognostic value of over 60 miRNAs in TNBC [104], highlighting the abundance of potentially promising exploratory avenues in this field. For example, low tumour miR-30a, miR-27a, and miR-301a dysregulation independently correlates with increased metastatic potential, and conversely, miR-30a overexpression curtails EMT and tumour cell invasion [105]. In the same analysis, miR expression levels correlated with overall outcomes, whereby underexpression of miR-30 and miR-374a/b was associated with inferior oncological outcomes, and increased miR-27a/b and miR-301a/b conferred inferior OS. Meanwhile, minimally invasive multi-miRNA prognostic signatures have been developed to predict recurrence and survival, such as the serum-based four-miRNA signature (miR-18b, miR-103, miR-107, miR-652) identified by Kleivi Sahlberg et al. in 2015 [106]. Efforts have also been made to identify patients who may not respond to NAT early, and miR-125b, miR-221, miR-222 and miR-199a-3p have been linked with chemoresistance [103,107,108]. Finally, miRNAs’ predictive value with respect to chemotoxicity has been defined, with reduced miR-195/miR-21 and increased miR-145/miR-10b predicting distinct side effects following systemic chemotherapy in a 2023 study [109]. Collectively, these findings underscore the potential of miRNAs not only to refine TNBC subclassification but also to serve as non-invasive biomarkers for diagnosis, prognosis, and treatment stratification.

### 4.4. miRNAs in Clinical Trials

Translational advances and the ongoing need for robust biomarkers in TNBC have driven the integration of miRNAs into clinical trials, evaluating their diagnostic, prognostic and therapeutic potential. The MODE-B study [110] provided valuable insights into miRNAs as a means of early, minimally-invasive TNBC diagnosis via liquid biopsy. Through PAXgene venous sampling, a 7-miRNA (miR-126–5p, miR-144–5p, miR-144–3p, miR-301a-3p, miR-126–3p, miR-101–3p, miR-664b–5p) signature was derived that distinguished early invasive TNBC with 84% sensitivity. miR-126–5p correlated most strongly with disease presence. This strong correlation was consistent with prior tissue-based findings from Paszek et al. [97], albeit interestingly, significantly reduced miR-126–5p levels were demonstrated in tissue compared to elevated blood levels in MODE-B. Moreover, MODE-B demonstrated that miRNA expression dynamics, rather than baseline levels, may predict treatment response: 321 miRNAs were deregulated pre- and post-chemotherapy, with upregulation of miR-34a-5p and reductions in miR-144–3p, miR-144–5p, miR-126–5p, and let-7d-5p being significant. The N-1 study [111] extended this principle by prospectively integrating miRNA microarray profiling into a neoadjuvant regimen of S-1 and docetaxel, finding that miR-215–5p expression was significantly higher in patients achieving pCR, highlighting the value of miRNA profiling in predicting neoadjuvant response.

Chemoresistance remains a defining challenge in TNBC, with up to 90% of agents eventually losing efficacy [112]. Beyond correlative studies, novel strategies for treatment-resistant disease are under investigation. Preclinically, formononetin use has been shown to modulate Paclitaxel resistance through miR-199a-3p downregulation [113]. The MRX34 phase I trial, a first-in-human study of a liposomal miR-34a mimic in refractory solid tumours including TNBC, provided proof-of-concept for miRNA-based therapeutics but was halted due to immune-mediated toxicities [114]. In GeparSixto (phase II) [50], pre-treatment exosomal miRNA expression was analysed in HER2-positive and TNBC patients. Among 224 TNBC cases, 17 miRNAs were deregulated compared with healthy controls, with miR-155 and miR-301 most strongly predicting pCR and miR-155 levels decreasing significantly post-treatment. Other miRNAs including miR-27a, miR-376a, and miR-376c normalised post-treatment, supporting their role as circulating biomarkers of response. Similarly, the NACATRINE trial [115] identified four dynamic plasma miRNAs predictive of pCR, further supporting miRNA-based treatment monitoring.

Prospectively, circulating and tissue-based miRNAs have the potential to inform therapeutic susceptibility and guide trial design. Previous work highlighting miRNAs’ tumour-suppressive effects, such as miR-199a-3p sensitising BRCA-mutated TNBC to PARP inhibitors, provides strong biological rationale for further combinatorial strategies in future studies [116]. Future studies, such as TARMAC (NCT04771871), aim to evaluate early treatment response. Moreover, biomarker-driven frameworks, as exemplified by the *I-SPY 2* trial [117], will enable real-time response-guided therapy allocation, ensuring patient-centred care remains at the fore of TNBC clinical investigation while integrating novel molecular indicators of response. miRNAs linked with TNBC are available in Table 3.

**Table 3:** miRNAs linked with TNBC.

miRNA	Tissue	Expression Change	Target/Pathway	Effect/Functional Note	Clinical Implication	Author (Year)
miR-10b, miR-145,	Whole blood	↑ miR-145; ↑ miR-10b	—	High miR-145 → higher risk of nausea/vomiting High miR-10b → higher risk of anaemia	Circulating predictors of GI toxicity (miR-145) and chemo-induced anaemia (miR-10b)	[109]
miR-18b, miR-103, miR-107, miR-652 (panel)	Serum	All ↑ in relapsing vs non-relapsing TNBC	EMT, DNA repair, TP53-linked pathways	High levels predict early recurrence and poor OS; strongest when combined into 4-miRNA signature	Non-invasive prognostic panel identifying high-risk TNBC at diagnosis	[106]
miR-21	Tumour/plasma	↑ highly expressed in TNBC	PTEN → PI3K/AKT activation	Promotes proliferation, survival, invasion; TNBC shows strong PTEN loss–miR-21 axis activation	Marker of aggressive TNBC biology; potential indicator for PI3K/AKT-targeted therapy sensitivity	[90,91]
miR-21, miR-16, miR-199a-5p	Plasma	All ↓ in TNBC vs. non-TNBC and controls	miR-21 → PTEN/ER-linked miR-16 → cyclin E/tumour suppressor miR-199a-5p → SRF/autophagy pathway	All three reduced in TNBC; levels rise post-surgery → tumour-load dependent. miR-199a-5p shows strongest diagnostic accuracy	Combined diagnostic panel for TNBC; miR-199a-5p strongest single biomarker; useful for early detection and monitoring	[103]
miR-21, miR-195	Whole blood	↓ miR-195; ↓ miR-21	—	Low miR-195 → higher risk of neutropenia Low miR-21 → higher risk of mucositis	Potential early markers for bone marrow suppression and mucosal toxicity during NAC	[109]
miR-27a/b	Tumour	↑ upregulated in TNBC	PTEN/PPAR pathways; CDC27 cell-cycle regulation	Promotes proliferation, invasion, and hormone-independent growth; contributes to tumour aggressiveness	High expression associated with worse OS and more aggressive clinical behaviour	[104]
miR-30	Tumour	↓ downregulated in TNBC	EMT suppression (E-cadherin ↑, N-cadherin/vimentin ↓)	Loss of miR-30 promotes EMT, invasion, and tumour progression; associated with more aggressive phenotype	Low miR-30 predicts poorer RFS and may identify biologically aggressive TNBC	[104]
miR-34a-5p	Tumour	↑ after chemotherapy (dynamic)	p53 pathway/tumour suppressive	Upregulated post-chemo; may reflect treatment effect	Possible dynamic biomarker of response	[97]
miR-34a-5p	Whole blood	↑ after chemotherapy (dynamic)	—	Upregulated post-chemo; may reflect treatment effect	Possible dynamic biomarker of response	[110]
miR-301a/b	Tumour	↑ upregulated in TNBC	CIP2A–PP2A oncogenic axis	Drives proliferation and invasion; enhances metastatic potential and supports tumour growth	Linked to more aggressive TNBC biology	[104]
miR-374a/b	Tumour	↓ downregulated in TNBC	CDKN2A (cell-cycle and tumour-suppressor regulation)	Reduced miR-374a/b expression promotes tumour progression and reduced genomic stability	Low levels predict shorter DFS and may mark more aggressive TNBC	[104]
miR-126–5p	Tumour	↓ after chemo (dynamic)	Angiogenesis/vascular links	Diagnostic and dynamic response marker	Diagnostic circulating signature; monitoring of response	[97]
miR-126–5p, miR-144–3p, miR-144–5p, let-7d-5p	Whole blood	↑ pre-NCT; normalise post-NCT	—	Show pattern before therapy that resolves after chemo	Chemotherapy-responsive markers for monitoring NCT effect	[110]
miR-126–5p; miR-144–3p/5p; miR-101–3p; miR-664b-5p (panel)	Whole blood	All ↑ in TNBC vs controls (pre-NCT)	—	7-miRNA diagnostic signature (84% sensitivity)	Diagnostic circulating signature for early TNBC	[110]
miR-155	Plasma	↑ higher in TNBC	SOCS1 → STAT3 activation	Elevated levels at diagnosis; decline after surgery/therapy; higher levels mark aggressive biology and high-risk features	Circulating risk marker	[94]
miR-181a	Tumour	↑ markedly upregulated in TNBC and metastatic tumours	Bim (↓) → anoikis resistance; ↑ Src/Akt/ERK signalling; TGF-β–driven EMT	Promotes EMT, migration, invasion, survival in suspension; enhances metastatic outgrowth in vivo; inhibition restores Bim and increases apoptosis	Predictive marker of metastasis and poor survival; potential therapeutic target in metastatic TNBC	[98]
miR-200 family (miR-200b/c) & miR-205	Tumour	↓ (EMT signature)	ZEB1/TGF-β → EMT control	Loss promotes EMT and metastasis; levels change with progression	Prognostic for metastasis/relapse; therapeutic targets	[99,100]
miR-215–5p	Tumour	↑ in pCR vs non-pCR	SOX9 suppression; anti-oncogenic regulatory axis	Higher baseline levels associated with chemosensitive TNBC and significantly enriched in patients achieving pCR	Potential predictive marker of NAC response	[111]
miR-498	Tumour	↑ upregulated in TNBC	BRCA1 (direct 3′UTR inhibition)	High miR-498 suppresses BRCA1; increases proliferation; inhibition restores BRCA1 and reduces TNBC cell growth (Hs578T)	Potential driver of BRCA1-low TNBC; biomarker of sporadic TNBC biology and possible therapeutic target	[95]

↑; upregulated, ↓; downregulated.

## 5. Immunotherapy

### 5.1. Current Role in Breast Cancer

Endocrine and HER2-targeted therapies have transformed outcomes in luminal and HER2+ breast cancers in recent years, whereas TNBC lacks such targets and historically carries poorer outcomes [10,48,118]. Immunotherapy has, therefore, represented a major therapeutic advance in TNBC, first in the metastatic setting and, more recently, in earlier disease [80]. Immune checkpoint inhibitors (ICIs) have shown robust response and are most often used with concurrent therapies to enhance antigen release, immune priming, and response durability [85,86,119]. In current practice, two ICIs are approved for treatment of breast cancer—both for TNBC. Pembrolizumab (anti-PD-1) is considered the gold standard in combination with chemotherapy for PD-L1-positive metastatic TNBC but also in early high-risk TNBC in conjunction with neoadjuvant chemotherapy, followed by adjuvant pembrolizumab [82,120]. Atezolizumab (anti-PD-L1) is recommended as an alternative first-line for unresectable locally advanced or metastatic PD-L1-positive TNBC in combination with nab-paclitaxel [120]. Presently, no checkpoint inhibitors are approved for ER+/HR+ or HER2+ disease, despite ongoing trial-based investigation.

### 5.2. Biomarkers

PD-L1 remains the principal clinical biomarker guiding ICI use in TNBC since foundational observations in 1992 [121,122]. IHC assays define its presence, albeit definitions vary based on assay: seminal pembrolizumab trials employ the 22C3 combined positive score (CPS), with greatest benefit at scores ≥ 10 [119,120], whereas atezolizumab validation studies utilised the SP142 IHC assay (≥1% immune-cell staining) [86]. While these therapies have had a transformative effect on outcomes in high-risk breast cancer, inherent challenges including response rate variation (reported from 15 to 60%) and tumour microenvironment (TME)-driven resistance further the pursuit of reliable markers for treatment susceptibility [123]. Additional candidates include tumour-infiltrating lymphocytes (TILs), where higher TIL ratio correlates with improved chemo-immunotherapy response, and cytotoxic T-lymphocyte-associated protein 4 (CTLA-4) expression, which is implicated in immune dysregulation in malignancy [124,125]. Tumour mutational burden (TMB)**,** homologous recombination deficiency (HRD)**,** and immune gene signatures show theoretical promise, though none are clinically validated [126].

### 5.3. Clinical Trials

Despite discrepancies in its measurement, PD-L1 remains the only approved biomarker in ICI breast cancer clinical trials, as utilised in the seminal KEYNOTE trial series, which established pembrolizumab as the benchmark ICI in TNBC. Early-phase studies (KEYNOTE-012, KEYNOTE-086) [127,128] established durable responses to monotherapy in metastatic PD-L1-positive TNBC, while KEYNOTE-355 [119] confirmed that pembrolizumab plus chemotherapy significantly improved survival in advanced TNBC with CPS ≥ 10. KEYNOTE-522 [85] extended these benefits to early-stage TNBC, demonstrating superior pCR and event-free survival with neoadjuvant pembrolizumab plus chemotherapy. Parallel IMpassion trials evaluated atezolizumab. IMpassion130 showed improved disease outcome in PD-L1-positive metastatic TNBC, though these results were not reproduced in the subsequent IMpassion131, leading to withdrawal of regulatory approval outside of Europe. Novel trials aim to augment response rates with the addition of PD-L1 inhibitors in ER+ (KEYNOTE-756 [129]/CheckMate 7FL [130]) and HER2+ (PANACEA [131]) subtypes, while dual-checkpoint approaches of PD-L1 and CTLA-4 modulators aim to overcome growing resistance profiles. Despite progress, response remains variable, underscoring the need for novel biomarkers for refined prediction of treatment response and resistance. Recently, miRNAs have shown some promise in this setting [18].

### 5.4. miRNAs and Immunotherapy

miRNAs are understood to play an important role in PD-L1 expression regulation. For example, miR-873 modulates the PD-1/PD-L1 axis, herein downregulating PD-L1 expression to reduce tumour proliferation in cancer. Additionally, miR-200 downregulates CD274 (the gene that encodes PD-L1) and subsequently PD-L1 [132], and miR-195/miR-497 have been shown to interact with CD274 in TNBC specifically [133]. Additionally, the RNA circFGFR4 modulates the miR-185–5p/CXCR4 axis to confer immune-evasion and anti-PD-1 therapy resistance in TNBC [134].

Furthermore, miRNAs have shown a promising role in sensitisation to ICIs. Zhang et al. demonstrated this in a breast cancer-specific preclinical model, identifying miR-149–3p as a key regulator of T-cell exhaustion in breast cancer. Using a murine 4T1 breast tumour model, the authors found that miR-149–3p was markedly downregulated in PD-1^+^ exhausted CD8^+^ T cells. Following transfection of CD8^+^ T cells with an miR-149–3p mimic, cell models exhibited reversal of the exhausted phenotype through downregulation of PD-1, reduced apoptosis and enhanced cytotoxic killing of breast tumour cells [135]. This research underscored the potential of miRNA in reinvigorating immune function to complement ICI agents while highlighting the fact that a single miRNA may target multiple checkpoint molecules. miR-149–3p concurrently regulated PD-1, TIM-3 and BTLA simultaneously, thereby restoring anti-tumour immune activity. Furthermore, miR-21–5p has been reported to be overexpressed in breast cancer, and suppression, in turn, reduces invasion of tumour cells [136,137]. Importantly, miR-21 has been implicated in both PD-1/PD-L1 axis and CTLA-4 modulation across oncological fields [91,101]. Although investigated preclinically to date, these biomarkers may prove crucial in therapeutic sensitisation and prolonging response durability as treatment resistance proves problematic in precision oncology.

## 6. Future Directions

Since their initial discovery in human tissue over two decades ago [138], miRNAs have undergone a remarkable translational journey from basic molecular regulators to clinically relevant biomarkers. Early cellular studies revealed their dysregulation in malignancy [139,140], and more recently, their capacity to classify tumours by molecular subtype has laid the groundwork for biomarker-driven oncology [141]. The subsequent identification of circulating miRNAs in blood and serum extended their utility beyond the confines of tumour tissue, introducing a minimally invasive means of disease detection and longitudinal monitoring [94,102,110,142]. Along with potential in diagnosis of early-stage breast cancer, circulating signatures have been shown to reflect treatment response, stratify risk, and capture resistance mechanisms across endocrine-responsive, HER2-positive, and triple-negative breast cancer [36,49,50,51,52,68,69,102,115,143,144]. Collectively, these findings illustrate miRNAs’ capacity to provide real-time insights into tumour dynamics while minimising procedural burden.

Despite strong biological rationale and promising early-phase findings, microRNAs have not yet been incorporated into routine clinical practice. This reflects several well-recognised challenges. There is considerable variability in how miRNAs are measured, including differences in sample type, laboratory techniques, and data normalisation, which limits reproducibility between studies. Much of the existing evidence remains exploratory, with a lack of large prospective studies validating their clinical utility. In addition, the biological complexity of miRNAs, whereby a single miRNA can regulate multiple targets and pathways, complicates interpretation and clinical application. Differences between circulating and tumour-derived miRNA profiles, as well as dynamic changes during treatment, further limit reliability as stable biomarkers. From a therapeutic perspective, challenges in targeted delivery and potential off-target effects have also slowed clinical translation. Together, these factors have contributed to the gap between promising experimental data and routine clinical implementation.

This work emphasises the potential of miRNA-based diagnostics and therapeutics across both tissue and liquid-biopsy settings while acknowledging the current lack of prospective, trial-based evidence and highlighting the need for further research to bridge this translational gap. The relationship between circulating and tumour-based miRNAs is of interest, as there is evidence to suggest an inverse correlation between blood and tissue miRNA [110,145], albeit this finding is inconsistent throughout the literature, with some work suggesting no relationship [146]. In the current paradigm, further elucidating this correlation may be of interest, with a view to enhancing minimally invasive and personalised cancer care.

While several microRNAs identified in this review (including miR-21, miR-155, miR-221, and members of the miR-200/205 family) are detected across all breast cancer subtypes, this overlap reflects both limited subtype specificity within breast cancer and a broader lack of cancer specificity. Many of these miRNAs are well-established “oncomiRs” dysregulated across multiple malignancies, including lung, colorectal, and hepatocellular cancers, where they regulate conserved pathways such as PI3K/AKT signalling, apoptosis, EMT, and immune modulation [144,147]. Within breast cancer, their presence across multiple subtypes likely reflects their role in regulating shared oncogenic processes, such as treatment resistance (miR-221) and EMT (miR-200/205), rather than simple redundancy [148,149]. Consequently, individual miRNAs are unlikely to provide sufficient discriminatory power on their own. Instead, combining multiple miRNAs into subtype-specific signatures, particularly when assessed over time, may offer greater clinical utility for diagnosis, prognosis, and predicting treatment response.

Looking ahead, miRNAs are poised for further incorporation into studies exploring their therapeutic potential. Recent evidence suggests that miRNA modulation may enhance the efficacy of emerging breast cancer vaccines, with combined miRNA vaccine strategies shown to strengthen antigen presentation, immune activation, and durable anti-tumour immunity, particularly relevant to triple-negative breast cancer where immunogenicity remains a therapeutic challenge [144]. Their capacity to act as therapeutic sensitisers by modulating pathways that influence response to targeted therapies is a further promising development in this field. By regulating key mediators of immune evasion, DNA repair, and apoptosis, including PD-L1, CTLA-4 and BRCA1-related networks, miRNAs can enhance tumour immunogenicity and restore susceptibility to checkpoint inhibition and other novel therapeutic strategies. Building on these advances, efforts are now returning miRNA therapeutics to clinical development. Emerging ligand-conjugated and nanoparticle-based delivery platforms are advancing miRNA mimics and anti-miRs back toward clinical evaluation, addressing the immune toxicity that halted earlier trials such as MRX34 [114,150]. As precision oncology evolves, integrating miRNA profiling within adaptive, biomarker-driven clinical trial frameworks will be essential to link molecular response with clinical endpoints, accelerate translation, and ultimately realise miRNAs’ dual potential as both biomarkers and therapeutic targets in breast cancer.

A major challenge remains in the absolute quantification of miRNAs in relevant clinical settings. PCR-based identification of the cycle threshold for abundance and measurement may vary, making their incorporation into studies challenging. However, their ubiquitous nature, role in multigene signalling, interference potential and ability to act as master regulators make them very enticing as future biomarkers and therapeutic targets.

## Data Availability

No new data were created or analysed in this study.
